# Comparison of disrespect maternity care among immediate postnatal women in health centre and hospital of Siltie Zone Southern region, Ethiopia 2020

**DOI:** 10.11604/pamj.2022.43.51.32737

**Published:** 2022-09-30

**Authors:** Abdulfeta Shafi, Mifta Redi

**Affiliations:** 1 Department of Statistics, College of Natural and Computational Science, University of Werabe, Werabe, Ethiopia

**Keywords:** Mistreatment, childbirth, pregnancy, delivery, abuse

## Abstract

**Introduction:**

disrespect and abuse during childbirth are interactions or facility conditions that local consensus seems to be humiliating or undignified. Physical abuse, non-consented clinical care, non-confidential care, non-dignified care, discrimination, abandonment, and detention in health facilities are the recent building blocks of maternal health. The objective was to compare the magnitude of disrespect for maternity care and associated factors among mothers during the immediate postpartum period at Siltie Zone health centre and hospital.

**Methods:**

institutional based cross-sectional study design using an interview-administered questionnaire used to measure the magnitude of disrespect and abuse during childbirth in a health facility. One randomly selected hospital and six health centres from three woredas were considered. A total sample of 420 was allocated to the health facilities according to the average number of delivery services provided per month. Proportion test, percentage and frequencies were used to show the relationship between the numbers of mistreatment between hospitals and health centres.

**Results:**

the overall mistreatment during childbirth and after birth is about 99%. Mistreatment in health centres is 98% and 100% in hospitals. The two categories of disrespect and abusive care namely deny the right to information 69% in hospitals and 42% in health centres. Secondly, Non-confidential care 51% in hospitals and 31% in the health centre are frequently shared categories of mistreatment during childbirth.

**Conclusion:**

mistreatment in hospitals and health centres is the same and very high. Abandonment of care, denial of right to information and non-confidential care has a significant difference in hospital and health centre.

## Introduction

A key component of the strategy to reduce maternal morbidity and mortality has been to increase rates of skilled birth attendance and facility-based childbirth. While the global skilled birth attendance rate rose by 12% in developing regions over the past two decades, almost one-third of women in these regions still deliver without a skilled birth attendant [1]. Increasing the proportion of women delivering in a health facility is challenging, as it requires comprehensive efforts to overcome sociocultural, economic, geographical, and infrastructural obstacles to reach facility-based care [2]. Furthermore, it requires efforts to improve both the coverage and quality of care provided to women at health facilities, including women´s rights to dignified and respectful care [3].

Every woman has the right to dignified, respectful sexual and reproductive health care, including during childbirth [4-6], as highlighted by the Universal Rights of Childbearing Women charter [6]. Therefore, mistreatment during childbirth can represent a violation of women´s fundamental human rights [7-11] and can serve as a powerful disincentive for women to seek care in facilities for their subsequent deliveries [2] and [12-14]. In September 2014, the World Health Organization statement called for greater research, action, advocacy and dialogue on this important public health issue, in order to ensure safe, timely, respectful care during childbirth for all women [3]. Likewise, respectful care is a key component of both the mother-baby-friendly birthing facilities initiative [15] and the WHO vision for quality of care for childbearing women and newborns [14]. Bowser and Hill published a landscape analysis exploring the evidence for disrespect and abuse in facility-based childbirth and proposed a seven-category model [15], which was designed to stimulate dialogue and an implementation research agenda, rather than provide a comprehensive review of the global evidence. Physical abuse, non-consented clinical care, non-confidential care, non-dignified care, discrimination, abandonment, and detention in health facilities [11] and have been a building block of recent work on this topic [16-19]. This study aims to compare the magnitude of disrespect for maternity care and associated factors among mothers during an immediate postpartum period at Siltie Zone health centre and hospital.

## Methods

### Operational definition

***Mistreatment:*** under this study women said to be mistreated if the women had experienced at least one of 24 indicators of disrespect classified into seven behavioural types based on Bowser and Hill classification [15].

### Methodology

***Study area, population and period:*** Siltie zone is one of the administrative zones in southern region located 173 km away from Addis Ababa. This zone has 4 hospitals (three primary hospitals and one comprehensive and specialized hospital) and 34 health centres. Institutional based cross-sectional study was conducted to assess the magnitude of disrespect and abusive maternity care among women who gave birth in public hospitals and health centres. The data collection period was from March 15, 2021 to April 30, in Siltie Zone, South Ethiopia. This study was done in three randomly selected woreda (Silti, Alicho wirirro and Ulbarag) and one randomly selected Kibet hospital. Two health centres were randomly selected in each woreda and a total of six health centres and one hospital were given consideration in this study.

***Inclusion and exclusion criteria:*** mothers who gave birth via elective or emergency caesarean section and were unable to communicate at the time of data collection were excluded. To maintain similarity between the services provided to study subjects between health centres and hospitals, rule out the effect of anaesthesia and minimize the time lapse between childbirth and the time of interview.

***Study design and sampling methods:*** institutional based cross-sectional study design was conducted. All women who were present health facility during data collection and at postpartum were included. A total of 419 women were randomly selected and interviewed.

***Data collection tool and producers:*** face-to-face interview questionnaire was used to collect data from women. The questionnaire was developed by reviewing different literatures.

***Sample size and sampling technique:*** a single population proportion formula was used to estimate sample size, with assumptions of 5% margin of error, 95% confidence, and a non-response rate of 10% and the proportion (p=0.469) of the prevalence of disrespect maternity care in previous related study in Sidama region 46.9%. A total sample of 420 was allocated to each health facility according to the average number of delivery services provided per month in the health facility. Kutere health center = 50, Qawaqoto Health center =48, Manahara Health center = 55, Kuno Health center 50, Karate Health center =66, Bilawanja Health center =50, and Kibat Hospital =104. Mothers were consecutively interviewed before being discharged from all health centres and a hospital until the desired sample size is achieved.


$$n=\frac{{\left(Z\alpha /2\right)}^{2}P\left(P\right)}{d^{2}}$$

Where n= total required sample size Z α/2= critical value 1.96 at 95% confidence interval P= 0.469, proportion of disrespect and abuse q=1-0.469=0.531 d = 5% 3.84*0.469*0.531 0.0025 = 383 with 10% of non-response rate the final sample size was 420.

### Study variable

***Outcome variables:*** the outcome variable for this study was experiences of mistreatment during childbirth based on 24 indicators classified into seven behavioural types. These classifications were Physical abuse, Verbal abuse, Non-consented care, Right to information, Non-confidential care, Abandonment of care and Discrimination [14]. Taking into consideration that participants have previously given birth, they will be instructed to answer these questions based on experiences during their most recent childbirth. Positively worded items were reverse coded for consistency where 1 referred to experienced mistreatment, and 0 to not experience. Total scores ranged from 0-24, where a higher score reflected a higher level of mistreatment.

***Independent variables:*** several variables were selected as factors associated with mistreatment. These factors were categorized as follows: (I) Socio-demographics: women´s age, Education, Sex of birth attendant, ethnicity, employment status/occupation and residence (II) Reproductive history: gravida (whether experienced childbirths before), (III) obstetric history: -ANC, number of pregnancies, place of delivery, time of delivery and companion presence, history of institutional delivery.

***Statistical methods:*** after data collection, each questionnaire was checked for completeness, consistency and clarity by data collector. Cleared data entered into SPSS software then imported to R-software, and Minitab and explored for missed values, outliers, and any inconsistencies. Descriptive statistics like percentage, graphs and descriptive summaries used to describe the study variables. Proportion of mistreatment and abuse during childbirth between health centres and hospitals were compared. P-value < 0.05 was considered statistically significant difference in all tests of significance.

**Ethical approval and participant consent:** ethical approval was obtained from Werabe University Review Board and an official letter was submitted to the public health institutions. The collected data was used for this research purpose only and kept with complete confidentiality. Verbal informed consent was obtained from the study participants and personal identifiers were excluded during the data collection to assure confidentiality.

**Funding:** this research was partially supported by Werabe University. University has no role in this paper other than funding.

## Results

Under this study response rate was 100%. However, during data cleaning four (4) individuals were removed due to poor quality and lack of full necessary information for our study, resulting final sample size of study to 419.

### Socio-demographic characteristics of mothers in Siltie Zone Six health and Kibet hospital, southern region, Ethiopia, 2021 (N=419)

Entirely 419 mothers participated in this study and have a mean of age 27.5 with a standard deviation of 6.003. About 98.8% of mothers were married and a very small percent (1.2%) accounted for single, divorced, and widowed. Most of the interviewed mothers (97.6%) are from the Siltie ethnic group. Nearly 69.5% of mothers were from rural areas and the rest (30.5%) were from urban areas. Approximately, 71.6% of mothers were homemakers and only 7.9% were government employed. Around 43.9% of respondents are illiterate and only 2.4% of mothers have degrees and above educational levels (Table 1).

**Table 1:** socio-demographic characteristics of mothers in Siltie Zone Seven health facilities, Southern region, Ethiopia, 2021 (N=419)

Variable	Category	Frequency	Percentage
**Marital status**	Married	414	98.8
	Single	2	0.5
	Divorced	2	0.5
	Widowed	1	0.2
		**419**	**100%**
**Ethnicity**	Siltie	409	97.6
	Amhara	3	0.7
	Oromo	1	0.2
	Other	6	1.4
		**419**	**100%**
**Residential area**	Rural	291	69.5
	Urban	128	30.5
		**419**	**100%**
**Educational level**	Illiterate	184	43.9
	Primary	143	34.1
	Secondary	39	9.3
	Preparatory	13	3.1
	Diploma	30	7.2
	Degree and above	10	2.4
		**419**	**100%**
**Occupation**	House wife	300	71.6
	Farmer	51	12.2
	Merchant	34	8.1
	Government	33	7.9
	Others	1	0.2
		**419**	**100%**

### Number of mistreatment during childbirth and after birth in Kibet hospital and health centre in Siltie zone, Southern region, Ethiopia 2021 (N=419)

In health centres, only 2% of mothers delivered during the study period get respected maternity care. However, in the hospital, 0% percent of mother delivered during the study period gets respected maternity care. Nearly, 27% of health centre-delivered mothers experienced one kind of disrespect during or after birth. Mothers experienced more than two kinds of disrespect accounts 48% in the health centre and 100% in hospitals (Table 2).

**Table 2:** number of mistreatment during childbirth and after birth in hospital and health centre in Siltie Zone, Southern region, Ethiopia 2021 (N=419)

		Health Center		Hospital		Total
S. No	Number of mistreatment	Frequency	Percentage	Frequency	Percentage	
1	0	5	2%	0	0%	5
2	1	85	27%	0	0%	85
3	2	73	23%	0	0%	73
4	>2	154	48%	102	100%	256
**Total**		317		102		419
**Percentage of mistreatment**		**98%**		**100%**		**98%**

### Comparison of Hospital and health centre disrespect and abuse during childbirth in Siltie zone, Southern region, Ethiopia 2021 (N=419)

The Table 3 summarizes the response of 102 mothers in hospital and 317 mothers in 6 health centres. The overall mistreatment during childbirth and after birth is about 99%. Mistreatment in health center 98% and 100% in hospital (Table 2). The two categories of disrespect and abusive care namely deny the right to information 69% in hospitals and 42% in health centres. Secondly, Non-confidential care 51% in hospitals and 31% in health centres are frequently shared categories of mistreatment during childbirth in all health facilities. The proportion of mistreatment in each category of disrespect and abuse during labour and after birth is almost similar in hospitals and health centres. However, Abandonment of care, denial of Right to information and non-confidential care has significant (p-value=0.000) difference in hospital and health centre. That is, Abandonment of care and denial of the Right to information is relatively higher in hospitals compared to health centres. In addition to this, Three subcategories of non-consented care and four subcategories of non-confidential care have significant differences in hospitals and health centres (p-value=0.00) (Table 3).

**Table 3:** comparison of hospital and health centre disrespect and abuse during childbirth in Siltie zone, Southern region, Ethiopia 2021 (N=419)

Variable	Category	Hospital		Health center		C.I& P-value	C.I &P-value
**Physical abuse**	Beating	12%	22%	4%	6%	(-0.16, 0.002) P-Value= 0.083	(-0.02, 0.006) P-Value= 0.268
	Slapping	10%		5%		(-0.14, -0.01) P-Value= 0.004	
	Push badly to change position	43%		10%		(-0.10, 0.018) P-Value =0.112	
	Pinch irritably	25%		5%		(-0.44, -0.23) P-Value= 0.00	
**Verbal abuse**	Pass insulting or degrading comments	29%	26%	4%	7%	(-0.28,-0.10) P-Value= 0.00	(-0.005, 0.02) P-Value= 0.21
	Harsh tone or shouting	56%		19%		(-0.34,-0.16) P-Value= 0.00	
	Abusive language	4%		3%		(-0.47,-0.26) P-Value= 0.00	
	Threatening for poor outcomes; inch irritably	17%		3%		(-0.13,-0.02) P-Value= 0.00	
**Non-consented care:**	Perform procedure without consent or Explain about the procedure to be used for delivery	67%	33%	17%	12%	(-0.21,-0.06) P-Value =0.00	(-0.03,0.01) P-Value = 0.321
	Deny choices regarding births	21%		18%		(-0.60,-0.4) P-Value= 0.000	
	coercion to undergo caesarean section;	0%		0%			
**Abandonment of care**	Abandon women during childbirth or afterward	57%	39%	4%	5%	(-0.63,-0.43) P-Value= 0.000	(0.03,0.07) P-Value = 0.000
	Ignore while asking pain relief/medication	54%		10%		(-0.54,-0.34) P-Value= 0.000	
	Delay birthing after deciding for operative procedure	6%		1%		(-0.09,-0.002) P-Value= 0.003	
**Discrimination**	Denial of service due to ethnicity	0%	0%	0%	1%		(-0.001, 0.008) P-Value = 0.179
	Denial of service due to lack of money	1%		1%			
**Right to information**	Share results/diagnosis of medical reports	44%	69%	45%	42%	(-0.09,0.12) P-Value= 0.817	(-0.18,-0.12) P-Value = 0.000
	Encourage to ask questions	88%		43%		(-0.53,-0.37) P-Value= 0.000	
	Regularly share progress of childbirth	67%		37%		(-0.40, -0.19) P-Value= 0.000	
	Threatening for poor outcomes; inch irritably	76%		43%		(-0.43,-0.24) P-Value = 0.000	
**Non confidential care**	Privacy during examination	56%	51%	23%	31%	(-0.43, -0.22) P-Value= 0.000	(0.08,0.13) P-Value= 0.00
	Cover woman while taking to and from labour room	61%		23%		(-0.49,-0.28) P-Value= 0.000	
	Assure woman for confidentiality of information	59%		23%		(-0.47,-0.26) P-Value= 0.000	
	Women-provider conversation overheard by others	28%		55%		(0.16, 0.37) P-Value= 0.000	
**Overall percentage per institution**		100%		98%			

## Discussion

This study was conducted in Siltie Zone one hospital and six health centres. Prevalence rate of mistreatment in hospitals is 100%. Again, the result was very high as compared as report in Siltie Zone hospital-based study [20]. This report is greater than study done in Jimma University Medical Centre 91.7% [21], greater than study done in Bishoftu general hospital 55.8% [22], greater than Injibara general hospital 71.8% [23], greater than study done in western Ethiopia three hospital and three health centres [24], Greater than Sidama zone hospital South Ethiopia report 46.9% [25]. The report was a little bit greater than study done in Pakistan health facilities and home-based delivery 97% [26]. The result of this study was higher than study done on respectful maternity care during labour and childbirth with associated factors in west show a zone in Oromiya region [27]. This discrepancy may be due to study setting, different study period, West Showa zone may have strong and integrated health system control. The other reason may be the scarcity of resources like the delivery rooms and women´s reaction/inspiration to the act of birth attendant staff. Respectful maternity care on physical harm, right to information, confidential care, dignity and respect, discrimination-free service, free from verbal abuse and free from abandonment percentages were higher than in this study [27]. All these categories of disrespect proportion are varying in Ethiopia [20-25].

**Limitation:** some studies have limitations that should be addressed in the paper. In this regard, the result of this study may not be generalized to the whole health facilities in Siltie zone. Since the comparison had been done in one hospital and six health centres.

## Conclusion

Mistreatment in hospitals and health centres is the same and very high. Abandonment of care, denial of Right to information and non-confidential care has a significant difference in hospital and health centre.

### What is known about this topic

- Hospital-based cross-sectional studies have been done in 2018 before and the prevalence rate of mistreatment and abusive care is 67.7%;
- Complicated labour, long stay in the hospital, the existence of a companion during birth and antenatal care service are determinants of mistreatment and abusive care in the study area.

### What this study adds

- The magnitude of mistreatment and abusive care in Siltie Zone is very high (99%); study done in the zone before focused only on hospitals and the prevalence rate was relatively too less;
- This study focuses on normal delivery (no induction, no caesarean section, and no vacuum-assisted delivery) cases in both health facilities to remove the effect of a different mode of delivery and keep exposed uniformity to mistreatment.
